# Veterinary telemedicine practicability: Analyzing Russian pet owners’ feedback

**DOI:** 10.14202/vetworld.2024.1184-1189

**Published:** 2024-05-28

**Authors:** Sergey Vladimirovich Akchurin, Hassane Benseghir, Fayssal Bouchemla, Irina Vladimirovna Akchurina, Sergey Vasilievich Fedotov, Georgiy Petrovitch Dyulger, Veronica Vladimirovna Dmitrieva

**Affiliations:** 1 Department of Veterinary Medicine, Russian State Agrarian University - Moscow Agricultural Academy Named after K.A. Timiryazev, Moscow, 127550, Russia; 2 Department of Microbiology, Faculty of Natural and Life Sciences, University of Batna, 05078, Algeria; 3 Department of Animal Disease, Veterinarian and Sanitarian Expertise, Faculty of Veterinary Medicine, Vavilov Saratov State University of Genetic, Biotechnology and Engineering Saratov, Russia

**Keywords:** payment behavior, respondents, survey, telecommunication, veterinary telemedicine

## Abstract

**Background and Aim:**

Previous research points to a growth rate of 17% for veterinary telemedicine. This study aimed to analyze pet owners’ attitudes, feasibility, and socioeconomic impacts of introducing this growth technique to a particular demographic.

**Materials and Methods:**

Five hundred population-representative respondents were utilized in the study. The ages ranged from 18 to 68 years. At the Russian State Agrarian University’s veterinary hospital, respondents (pet owners) made contact (either in person or remotely). The survey inquired about participants’ personal information, their pets, and veterinary telemedicine. Russia uses the ruble, issued by the Bank of Russia, as its currency. The required sample size of 385 for this study was determined using the Q test to ensure feasibility.

**Results:**

79.2% of the participants had a positive outlook on telemedicine. Every fifth applicant turned down telemedicine, opting instead for personal vet appointments. 53.8% of respondents with prices under $14 were willing to pay for the service, whereas 17.8% (89 people) outright rejected it, and 93.8% of the paid customers belonged to the age group of 18–28. Pet owners with chronically ill animals merit special consideration.

**Conclusion:**

Pet owners are generally open to veterinary telemedicine, but it remains underutilized. The study reveals directions for optimizing veterinary telemedicine and enhancing client and patient satisfaction. Despite limitations (less access to respondents/telemedicine), future approach is to investigate variables and invariable factors affecting this process.

## Introduction

Telemedicine’s use in veterinary care has surged since the pandemic. In their book “Blue Ocean Strategy” [1], Chan and Mauborgne highlight intense market competition between conventional and innovative goods. According to current trends, the future of veterinary telemedicine is expected to expand considerably. The estimated global veterinary telemedicine market worth was $107.85 million in 2021 and is projected to hit $510.4 million by 2030, expanding at a compound annual growth rate of 17.69% [2].

The Canadian Veterinary Medical Association defines telemedicine as providing remote veterinary advice and treatment based on non-physical diagnosis using telecommunications technology (as per the current study [3], this definition of telemedicine involves no physical examination). Several ways have shown the need for this service.

1. Communication technologies, the Internet, and software advancements have enabled interactive opportunities between pet owners and veterinarians [4–6]
2. Customers benefit more from purchasing and making payments online in the e-commerce sector [7]. The pandemic fueled a significant increase in both e-commerce and telemedicine [7, 8]
3. The availability of wearable devices and biosensors enhances remote medical diagnosis by enabling real-time measurement of body temperature, heart rate, and other health-related factors [9–11]
4. It is most efficient for transporting large animals, saving significant time and effort. It is accessible in rural areas, enabling prompt attention to health emergencies [12–14]
5. Veterinarians find this service to be effortless, lucrative, satisfying to customers, and a means to lighten junior veterinarians’ workload [15, 16].

Among 155 pet owners polled [17], favorable views on telemedicine technologies, especially for emergencies, were reported. The age groups 18–29 and 30–44 showed a greater interest in veterinary telemedicine than the older age groups (45–59 and 60–75). According to the survey, millennial pet owners plan to pay an average of $40 for this service [17] (note that “plan to pay” implies readiness to spend). Cary and Massecar [18] raise concerns about a lack of awareness and access to existing technology. Telemedicine’s stress reduction benefits for pets outweighed the concerns of no clinical examinations and potential misdiagnosis among pet owners [19]. During this interval, some pet owners opted for telemedicine, utilizing techniques such as telecommunications and emails for their veterinary consultations [19].

Although the use of telemedicine services is increasing, there remains a scarcity of client feedback data [8, 19]. Related parties struggle to create an approach that guarantees high-quality telemedicine service.

To effectively serve pet owners, it is essential to conduct more research and clarify ambiguous aspects of past methods regarding this service.

This study aims to examine pet owners’ perspectives on veterinary telemedicine.

1. Assessing the mindset of pet owners regarding the application of telemedicine technologies
2. Assessing the effectiveness of tele-veterinary practices
3. Emphasize the health concerns that can be effectively managed through remote means
4. Conducting research to determine which telemedicine services for pet owners are willing to pay for
5. Identifying the influencing factors on payment attitudes and determining if these can be altered for chronic illnesses undergoing prolonged care.

## Materials and Methods

### Ethical approval

Since this study was analytical in nature, it was exempted from Ethical Committee approval; respondents’ written consents were obtained, and their data were processed anonymously per General Data Protection Regulation guidelines.

### Study period and location

The survey was conducted from January 2022 to March 2022. The study population comprised pet owners (respondents) who communicated with the Moscow, Russia Veterinary Hospital at the Russian State Agrarian University – Moscow Timiryazev Agricultural Academy.

### Study design

About 500 respondents took part in the survey. The age range varied from 18 to 68 years old. Based on the 2020 census data, the study sample was structured to match the age distribution of the Russian population. Survey questions included protected personal data, demographics, pet-related details (gender, age, animal type, and chronic diseases), and evaluations of veterinary telemedicine. Participants were queried about their experiences with veterinary services, their willingness to pay for telemedicine, and potential health concerns that could be managed remotely. The survey was carried out in Russian. Pet owners set their price range based on the Russian ruble exchange rate by the Bank of Russia on December 21, 2023. The survey’s price range was set based on Russian veterinary telemedicine market analysis.

### Data collection

The survey contained a mix of single-answer, multiple-choice, and open-ended questions. Both paper and electronic versions of the study were compiled with its data. The respondents could answer the questionnaire either partially or fully. The electronic form was uploaded to Google Forms [20].

### Statistical analysis

Using the Q test, we determined the appropriate study size to ensure statistical precision and confidence within an acceptable range. The Q formula can be written as: # original text: The Q formula is expressed as follows:

n_0_ = Z^2^Pq/e^2^

Where: e: Precision level; p: Sample proportion; q = 1 p, z: Statistic coefficient (from Z table) [21].

The authors aimed to identify the smallest sample size for maximum variability. With a 95% confidence level, a minimum feasible sample size of 385 can be obtained by assuming a population proportion of 0.5 from the half of clients who agreed to be interviewed. Descriptive statistics were calculated using Microsoft Excel on the spreadsheet. The study involved participants from the University Veterinary Hospital, either in person or virtually.

## Results

### Demographic categories

About 500 people took part in the survey. The classes were defined based on 10-year age intervals, ranging from 18 to 68 years old. The ages of the participants are shown in Table-1. It has been revealed that demographic categories 29–38 and 39–48 represent almost half of the respondents, whereas other age classes share the other half. Nearly all the participants were females (n = 382, 76.4%), whereas males represented merely a quarter (23.6%). A total of 309 of the participants were cat owners (61.8%) and 293 were dog owners (58.6%).

**Table 1:** Age classes’ distribution of respondents.

Age range/years	No. of respondents	Percentage
18–28	81	16.2
29–38	122	24.4
39–48	107	21.4
49–58	93	18.6
59–68	97	19.4

Besides meerkats, gerbils, magpies, snakes, toads, lizards, and starlings, as well as chickens and rabbits, visitors did not typically come to our hospital for those animals. The pets not observed or examined in this study were excluded. 160 interviewees (32%) owned multiple pets. One in every four pet owners reported having both a cat and a dog. Table-2 includes specifics on the number and variety of pets owned by each respondent.

**Table 2:** Distribution of pet type per respondent.

Pet type	No. of pet owner	No. of pet owner - %
Cat	309	61.8
Dog	293	58.6
Parrot	30	6
Rat	12	2.4
Horse	9	1.8
Cow	8	1.6
Guinea pig	8	1.6
Aquarium fish	5	1.0
Turtle	4	0.8
Ferret	3	0.6
Hamster	2	0.4
Gecko	2	0.4
Spiders	2	0.4
Snails	2	0.4
Chinchilla	2	0.4
Pig	2	0.4

### Attitude toward telemedicine

**Figure-1:**
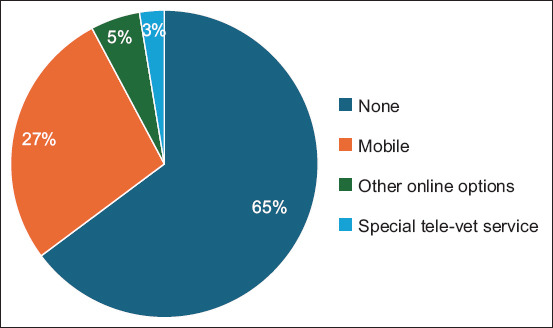
The distribution of respondents according to their experience with Veterinary Telemedicine services.

We ensure equal awareness of telemedicine among all participants. Based on the survey results, we intended to evaluate pet owners’ past encounters with this service. According to the obtained data, an overwhelming number of the respondents replied that they had no previous experience with veterinary telemedicine (n = 324, 64.8%), and merely 35.2% (n = 176) of them had experienced consulting their veterinarian through messenger and/or telephone. Tele-veterinary services were used only in 2.6% (n = 13) of the total, where special medical devices were provided by veterinary organizations to pet owners (Figure-1).

**Figure-2:**
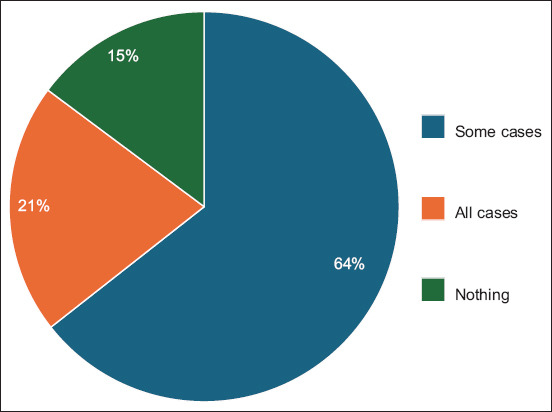
Respondents’ Feedback on health issues that can be solved remotely

64.4% of the respondents (n = 344) expressed a positive attitude toward telemedicine, intending to use it for case-dependent health issues, whereas 14.8% (n = 74) believed that all issues could be addressed remotely. 79.2% of the 418 respondents showed a positive attitude toward veterinary telemedicine. One in five respondents refused telemedicine, preferring face-to-face consultations (Figure-2).

### Hierarchy of health-related issues to be addressed in telemedicine

The questionnaire inquired about the specific veterinary issues individuals would prefer to discuss remotely. In this section, responses varied from full agreement to complete refusal. The necessity for telemedicine, as considered by the participants, is detailed in Table-3, including the specifics of when and how this need arose.

**Table 3:** Respondents’ preferences for approaching vet-telemedicine.

Percentage	Respondents’ telemedicine service preferences
67.4	Full channel for communication
42.4	Receive prescriptions
42	When pet gets sick
36.4	Seeking referrals
34.8	Updates
31.4	Ordering medications
30.2	Re-issuing prescription
27.6	Follow-ups checks
26.2	Health parameter monitoring
25.4	Information about disease/treatment
18	Reminders only
1.6	None

67.4% preferred to communicate with the veterinarian about their pets’ health issues, whereas 42% consulted the veterinarian only when problems arose or requested prescriptions. These options best reflect the collective preferences of the respondents. Approximately one-third of the participants engaged in activities such as seeking referrals, updates, monitoring, ordering medicines, reissuing prescriptions, scheduling follow-ups, and obtaining information about their pet’s illness and treatment. 1.6% of the respondents, aged 59 and older, opted for a one-choice answer and insisted on physically visiting our hospital.

### Socioeconomic patterns significantly influence the adoption and utilization of telemedicine services

Telemedicine, a recent development within E-commerce, expedites diagnostic processes but requires payment. Considering the economic implications, we inquired about the client’s stance on financing the mentioned services. We asked clients to classify tele-veterinary services based on their willingness to pay. While the findings were accepted by most participants, not all services received approval. Table-4 illustrates pet owners’ willingness to pay for various tele-veterinary services.

**Table 4:** Payment behavior toward different modalities of tele-veterinary services.

Percentages	Most likely to be paid telemedicine services
38.4	Full channel for communication
12	Medical prescription
30.6	When pet gets sick
11	Organize referrals
11.4	Updates
32.8	Ordering medications
12	Re-issuing prescription
25.4	Follow-ups checks
10.8	Consultation with diagnosis
11.2	Information about disease/treatment
7.6	Reminders only
21.8	None

Respondents’ feedback moderately varied, and the overall look can be divided into two categories: Those, who will to pay and other that object paying.

Full remote associability, follow-up checks, and ordering medications are commonly paid services, whereas referrals, reminders, and prescriptions have a lower profile in terms of payment. 21.8% of respondents believed that telemedicine should be free, whereas the rest preferred to pay for distinct remote services.

**Figure-3:**
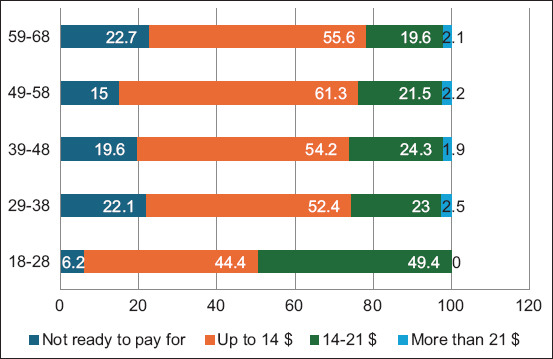
Age group distribution of payment behavior toward tele-veterinarian service.

Based on the data in Table-4, we determine the average cost respondents will accept for the proposed services. Figure-3 represents the outcomes categorized into the following payment classes: not ready to pay, $0-$14, $14-$21, and over $21.

Most pet owners (n = 269, 53.8%) demonstrated their willingness to pay for any service, provided the cost would not exceed $14, whereas 26.6% were ready to spend “14–21 $” and only 1.8% “more than 21 $.” 82.2% of respondents are willing to pay for telemedicine services. 93.8% of respondents fell into the 18–28 age group, whereas 85% were in the 49–58 age group. Older respondents show a more unified answer distribution.

### Disease chronicity and telemedicine

This study investigated the association between disease chronicity and telemedicine’s periodic payments. 79.8% of survey respondents reported no chronic diseases for their pets, suggesting that about 20.2% might benefit from telemedicine.

We assessed how long-term illness affected the owner’s readiness to purchase online vet care. 91.1% of pet owners with chronic disease were willing to pay compared to 82.2% of all participants. The longer the disease chronicity, the more positive the attitude toward vet-telemedicine. Pet owners with chronically ill pets face longer telemedicine journeys and higher expenses. The following shows the average distribution of payments made by pet owners with chronic diseases.

- Not ready to pay – 8.9%
- Pay up to 14 $ – 57.4%
- Pay interval 14–21 $ – 31.7%
- More than 21 $ – 2%.

## Discussion

To deliver excellent veterinary services through telemedicine, it is essential that we understand our client base, their expectations, and associated costs. 38.9% of telemedicine service cases consist of follow-ups for ongoing clients, 22.1% involve phone advice, and 20% entail professional advice [16]. It is illegal to provide remote diagnoses and medication prescriptions for animals. If the attending veterinarian has previously seen the pet, they may be exempt from the requirement to conduct a new examination.

This study bolsters existing information on pet owners’ perspectives toward veterinary telemedicine. The essential outcomes in this matter are as follows:

1. 64.8% of respondents were inexperienced with veterinary telemedicine services (n = 324). The comparable result was found in the Veterinary Innovation Council’s findings in 2017 [18]. Most respondents preferred consulting their veterinarian online for telemedicine services. A recent study by Caney *et al*. [19] produced results similar to ours. Regardless of population characteristics or geographical patterns, attitudes toward telemedicine may be similar
2. 2.6% of pet owners have employed special tele-veterinary devices, likely due to insufficient promotion. Pet owners’ low awareness causes uncertainty regarding veterinary telemedicine’s necessity. Pet owners need to be educated about their rights and responsibilities. An attending veterinarian can remotely prescribe or diagnose health issues, but an unknown veterinarian can only offer general advice. Enhancing the perception, awareness, and understanding of telemedicine use and implementation of marketing strategies within veterinary technology industries is essential [16, 17, 22]
3. 79.2% of respondents were open to resolving their health issues remotely, yet recognized the restriction on the scope of veterinary issues that could be addressed online. Ilukor *et al*. [5], Garner [15], Google Forms Platform [20], Gyles [22], and Becker *et al*. [23] noted that most pet owners are open to utilizing veterinary telemedicine. The preferences for telemedicine use varied among respondents with no consistent features identified
4. Telemedicine services with specific payment behaviors tend to be avoided by pet owners. 67.4% of pet owners prefer having a consultation channel with their veterinarian, but just 38.4% agree to payment terms. Registered clients within a clinic are entitled to telemedicine services, but the payment methods are subject to marketing decisions
5. The majority of respondents indicated the upcoming services that they were willing to pay for:
  - Allow consultant communication with the attending clinician
  - Consulting a veterinarian on call (outside office hours)
  - Order home delivery of medication and veterinary products
  - Carry out remote follow-up assessments.
6. 82.2% of respondents agreed to pay for veterinary telemedicine, with 57.4% setting a threshold of $14 for the payment. The detail provided here contrasts with C’s acquisition. According to Hawk [17], pet owners might be willing to pay an average of $40 for a telemedicine consultation. The difference can be explained by the telemedicine market’s value, currency fluctuations, and the type of service in each country.
  In the age group 18–28, 93.8% of pet owners pay for the service, and half of them (49.4%) pay an average of $14–$21. The millennial generation is known for extensive Internet surfing using various gadgets. Pet owners now prefer consulting veterinarians online.
  91.1% of pet owners with chronic diseases expressed readiness to pay for veterinary telemedicine.
7. 1 in 4 respondents desire a device to track their pet’s veterinary health data. The marketing and accessibility of these telemedicine tools need improvement. With the latest technological advancements, there will be an increase in telemedicine users.

## Conclusion

Despite a positive reception, veterinary telemedicine services have not effectively reached most pet owners. Population characteristics and similarities can impact attitudes toward telemedicine regardless of geographical patterns. Adhere to both national and international veterinary ethics and regulations in accommodating respondents’ preferences. The difference in payment rates for telemedicine is determined by the value of the market in each country, currency, and type of service. The youngest pet owners and disease chronicity are the most receptive to telemedicine. Telemedicine requires more promotion and understanding in the market. The upcoming technological advancements are projected to bring about an increase in telemedicine users. Future research could investigate correlations between variables affecting veterinary teleconsultation, namely service types (consultation, prescription, and sales), delivery methods (telephone, video, and email), cost, veterinarian reputation, and legal terms.

## Authors’ Contributions

SVA and FB: Planned and designed the study. IVA and SVF: Organized and supervised the fieldwork. HB, GPD, and VVD: Drafted the manuscript. SVA, HB, and FB: Analyzed data and developed the main body text. GPD and VVD: Addressed most of the discussion. SVF and IVA: Helped with the material and methods section. All authors have read, reviewed, and approved the final manuscript.
